# A Neuron‐Glia Co‐culture System for Studying Intercellular Lipid Transport

**DOI:** 10.1002/cpcb.95

**Published:** 2019-08-09

**Authors:** Maria S. Ioannou, Zhe Liu, Jennifer Lippincott‐Schwartz

**Affiliations:** ^1^ Department of Physiology University of Alberta Edmonton Alberta Canada; ^2^ Neuroscience and Mental Health Institute Edmonton Alberta Canada; ^3^ Janelia Research Campus Howard Hughes Medical Institute Ashburn Virginia

**Keywords:** apolipoproteins, fatty acids, intercellular transport, lipid transfer, primary neuron astrocyte cultures, sandwich assay

## Abstract

Neurons and glia operate in a highly coordinated fashion in the brain. Although glial cells have long been known to supply lipids to neurons via lipoprotein particles, new evidence reveals that lipid transport between neurons and glia is bidirectional. Here, we describe a co‐culture system to study transfer of lipids and lipid‐associated proteins from neurons to glia. The assay entails culturing neurons and glia on separate coverslips, pulsing the neurons with fluorescently labeled fatty acids, and then incubating the coverslips together. As astrocytes internalize and store neuron‐derived fatty acids in lipid droplets, analyzing the number, size, and fluorescence intensity of lipid droplets containing the fluorescent fatty acids provides an easy and quantifiable measure of fatty acid transport. © 2019 The Authors.

## INTRODUCTION

Coupling of lipid metabolism between neurons and glia is an important area of neuroscience research. Astrocytes and microglia have long been known to supply neurons with lipids and cholesterol, which neurons use to build and repair membranes, via lipoprotein particles. Recent work has revealed that under oxidative stress, neurons transfer lipids to lipid droplets in astrocytes via lipid particles (Ioannou et al., 2019). As lipid dysfunction is a common theme in neurodegenerative disease and injury such as stroke, simple assays to quantify lipid transfer from neurons to glia are critical for elucidating the mechanisms involved.

**Figure 1:**
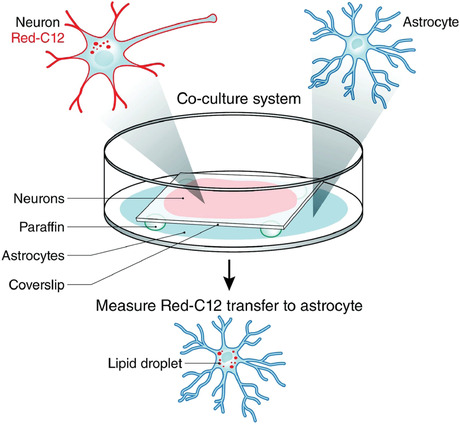
Schematic of fatty acid transfer assay. Neurons are incubated with Red‐C12 overnight and then incubated with glia on separate coverslips for 4 hr. Glia are fixed, and the appearance of Red‐C12 in astrocytic lipid droplets is imaged and quantified. This figure is reproduced from Ioannou et al. (2019).

Here, we report step‐by‐step protocols for performing assays of lipid transfer from neurons to glia (Ioannou et al., 2019). We first describe culturing of primary neurons and glia from postnatal rat pups (Basic Protocol 1) on separate glass coverslips (Support Protocol 1). The neurons are loaded with commercially available fluorescently labeled fatty acids and then incubated with astrocytes in a “sandwich” co‐culture system where neurons do not physically contact glia (Basic Protocol 2). Fatty acid transfer can be measured by imaging the appearance of fluorescently labeled fatty acids in the glial cells (Fig. 1). We include a detailed protocol for quantification of fatty acid transport using ImageJ (Support Protocol 4). Finally, we provide related protocols for labeling cells with cell type–specific markers or markers for related organelles, such as lipid droplets (Support Protocol 2), and for controlling for phagocytosis and cellular debris (Support Protocol 3). Although our protocol follows fatty acids delivered from neurons to glia, this assay in principle can be used to study lipid transport in the reverse direction (from glia to neurons) or between any two types of cells.

## Basic Protocol 1 CULTURING OF PRIMARY HIPPOCAMPAL NEURONS AND GLIA

This protocol describes how to culture primary hippocampal neurons and mixed glial cells from postnatal rat pups. The neuronal culture protocol, modified from a prior reference (Beaudoin et al., 2012), allows for culturing neurons at low densities without an astrocytic feeder layer. Glial cells are plated on separate coverslips. Although the viability and yield when culturing rat cells are higher, this protocol can also be used to culture postnatal mouse cells, which may be useful to assay fatty acid transfer in cells from genetically modified animals.

### Materials

70% (v/v) ethanol (optional)
Neuron medium (see recipe)
Plating/glia medium (see recipe)
Dissection medium (see recipe)
Papain (PDS Kit, Papain Vial; Worthington Biochemical, LK003178)
P0 rat pups
0.4% trypan blue solution (Gibco, 15250061)
Cytosine arabinoside (AraC; Sigma‐Aldrich, C1768)

Dissection instruments:
  Sharp surgical scissors (Fine Science Tools, 14001‐16)
  Extra‐fine Bonn scissors (Fine Science Tools, 14084‐08)
  Two Dumont‐style #3 forceps (Fine Science Tools, 11231‐30)
  Stainless steel laboratory spoon (VWR, 89259‐968)
  Iris spatula, 12 cm (Fine Science Tools, 10092‐12)
  Two Dumont‐style #5 forceps (Fine Science Tools, 11252‐30)
  Disposable scalpel (#11, VWR, 100499‐580)
Autoclave (optional)
6‐well cell culture plates
Poly‐d‐lysine–coated coverslips (see Support Protocol 1)
10‐cm cell culture plates or petri dishes
60‐mm cell culture plates
Dissection microscope with light source
Cut 1‐ml pipet tips
Sterile scissors
15‐ and 50‐ml conical tubes
37°C water bath
70‐µm nylon cell strainer (Corning, 431750)
Microcentrifuge tube
Hemocytometer
Standard tabletop centrifuge

*NOTE*: All solutions and equipment coming into contact with cells must be sterile, and proper sterile technique should be used accordingly.

*NOTE*: All culture incubations are performed in a humidified 37°C, 5% CO_2_ incubator unless otherwise specified.

### Prepare reagents for culture

1 Sterilize dissection instruments by autoclaving or rinsing with 70% ethanol.
2 Warm neuron medium and plating/glia medium to 37°C before use. Chill dissection medium on ice.
  Even if you are only growing neurons, you will still need to use glia medium, which contains serum, to inactivate the papain solution used to dissociate the tissue.
3 Add 2 ml pre‐warmed neuron or glia medium (depending on which cell type is to be cultured) to each well of 6‐well cell culture plates containing poly‐d‐lysine–coated coverslips. Incubate at 37°C before starting dissections to allow the temperature to equilibrate.
4 Make 2× papain solution by dissolving one vial of papain (∼120 U enzyme for 10 pups) in 5 ml ice‐cold dissection medium. Keep on ice.
  Although it is best to make this solution fresh immediately before use, 2× papain can be stored for up to 1 week at 4°C.

### Remove hippocampi from P0 rat pups

5 Fill 10‐cm cell culture plates or petri dishes with ice, use lids to pack the ice down flat, and then remove lids. Prepare 60‐mm cell culture plates containing ice‐cold dissection medium and place on ice packs.
  Plan to use one 60‐mm plate per two brains.
6 Euthanize P0 rat pups by decapitation with sharp surgical scissors.
7 Hold each head from the sides. Starting at the base of the brain, using extra‐fine Bonn scissors, cut skull along the center, toward the front of the head. Be careful not to cut brain itself. Make two small cuts through skull, away from the initial incision.
8 Using Dumont‐style #3 forceps, carefully peel skull off and to the sides.
9 Using a stainless steel laboratory spoon, scoop out brain and place it in a 60‐mm plate containing ice‐cold dissection medium (see step 5). Repeat steps 6 to 9 to collect the desired number of brains. Collect two brains per 60‐mm plate.
10 Replace dissection medium in each 60‐mm plate with fresh ice‐cold dissection medium.
  This step helps wash away some of the blood and damaged tissue from the brains. The brains need to be submerged in medium at all times.
  The #3 forceps and spoon are now considered “dirty” and should not be used for any of the following steps.
11 Prepare a fresh ice pack (see step 5) to be used during dissection: again, fill a 10‐cm cell culture plate or petri dish with ice, but keep lid on this time. Place one 60‐mm plate containing two brains on top of ice pack.
  Keeping the lid off the ice pack will keep your samples colder but will cause the 60‐mm plate to slide around, making the dissection more difficult.
12 Place ice‐packed plate on the stage of a dissection microscope with a light source. Position first brain upright and hold it in place by gently gripping cerebellum using Dumont‐style #3 forceps.
  We find 0.5× magnification to be optimal for this step.
13 Gently run an iris spatula between two hemispheres of the cortex to separate them. Use convex side of the spatula to gently open one hemisphere so that the hippocampus faces upward.
  The hippocampus is the C‐shaped structure that runs parallel to the cortex.
14 With the hippocampus facing up, place spatula between the hippocampus and the cortex. Using the spatula, separate tissue where the hippocampus meets the cortex to remove the hippocampus.
15 Using a 1‐ml pipet tip that has been cut with sterile scissors to make the opening wider, transfer hippocampus in a new 60‐mm cell culture plate containing fresh ice‐cold dissection medium on an open ice pack (see step 5).
16 Collect all remaining hippocampi before moving on to the next step.
17 Under the dissection microscope (2× magnification), turn hippocampus, with the convex side facing up. Using Dumont‐style #5 forceps, pin down hippocampus. Using a second pair of #5 forceps, gently peel off meninges.
  Complete removal of the meninges is critical to prevent growth of non‐neuronal cells. It is best to peel the meninges off as a single piece if possible. Meninges can be very sticky. Be careful not to damage the hippocampal tissue at this stage.
18 Using #5 forceps and a disposable scalpel, cut hippocampus into four pieces. Using a cut 1‐ml pipet tip (see step 15), transfer hippocampal pieces into a 15‐ml conical tube containing fresh cold dissection medium.
  Hippocampi from multiple animals can be pooled in one tube.
19 Warm 2× papain solution from step 4 in a 37°C water bath.
20 Wash hippocampal pieces three times with 5 ml cold dissection medium. To do this, allow pieces to fall to the bottom of the tube and aspirate dissection medium with a 1‐ml pipet.
  Make sure to remove any fragments of meninges/debris. They are not as heavy as the hippocampal pieces and will take longer to settle to the bottom of the tube during the washes. It may be helpful to gently disturb the settled hippocampal pieces so that the remaining meninges/debris are resuspended and can be effectively washed away.
21 Resuspend washed hippocampal pieces in 5 ml dissection medium. Then, add 5 ml of pre‐warmed 2× papain solution from step 19. Incubate in 37°C water bath for 20 min.

### Dissociate and plate neurons and/or glia

22 Using a 1‐ml pipet, slowly remove dissection medium containing papain. Be careful not to aspirate tissue, as it will be extremely sticky at this point. Wash two times with 5 ml plating/glia medium.
23 Resuspend tissue in 3 ml glia medium. Gently triturate tissue by pipetting up and down, first 10 times with a 10‐ml pipet and then 10 times with a 1‐ml pipet tip.
24 Assemble a 70‐µm nylon cell strainer over a 50‐ml conical tube. Pass dissociated cells through the cell strainer. Rinse 15‐ml conical tube with an additional 5 ml glia medium to collect any remaining cells that stuck to the tube and pass that medium through cell strainer as well.
25 To count the cells and assess their viability, transfer 10 µl cells to a microcentrifuge tube and add 10 µl of 0.4% trypan blue solution. Count cells using a hemocytometer.
  Viable cells are colorless, whereas dead and damaged cells are blue.
26 Split suspended cells from step 24 into two 15‐ml conical tubes. Centrifuge 5 min at 150 × *g*.
27 Resuspend cells in either neuron medium or glia medium, depending on the cells to be grown, and plate desired number of cells onto the prepared coverslips in 6‐well plates (see step 3).
  Generally, the desired number of cells per coverslip is 2 × 10^5^ for neuron cultures or 1.5 × 10^5^ for glial cultures. Cells should not be kept in suspension for too long, as the cells clump together and the viability can be compromised.
  Neurons secrete growth factors that support the health of the culture. For this reason, plating neurons too sparsely may negatively affect the health of the culture. However, plating neurons too densely will result in a higher number of contaminating astrocytes in the neuron culture, even in the presence of AraC.
  Glia will divide, and therefore, more or fewer cells may be plated depending on the timing of the experiment. One consideration is that when glia are plated more sparsely, a larger number of microglia will grow.
28 A total of 6 hr after plating the cells, replace approximately half of the medium by gently removing 1 ml medium from each well and adding 1 ml fresh pre‐warmed medium to the side of the well.
  The cells will be weakly attached at this point, so be careful not to detach the cells.
29 The next day, completely replace medium in each well with 2 ml neuron or glia medium.
30 To reduce the growth of glia in neuronal cultures, add 2 µM AraC to culture medium 2 days after plating (see step 27).
  This is critical to study lipids originating in neurons. Both astrocytes and microglia secrete lipoprotein particles, and therefore, it is important to have neuron cultures that are as pure as possible. This may not be necessary if you wish to study protein transfer and will express a protein in neurons using a neuron‐specific promoter.
31 Replace half of the medium every 3 to 4 days with neuron or glia medium, depending on type of cell being grown.
  If AraC was added, this will dilute out over time.
  Although mixed glial cultures primarily contain astrocytes, microglia and oligodendrocytes are also present. We recommend immunostaining with antibodies to identify the cell type involved in internalizing transferred lipid or protein (see Support Protocol 2). Pure glial cultures can be achieved by a variety of methods. Both shaking and mild trypsinization protocols take advantage of the varying adherence strengths of different cell types (Jana, Jana, Pal, & Pahan, 2007; Saura, Tusell, & Serratosa, 2003). These protocols are cost and time effective and easy to employ; however, they have a small degree of cell contamination. If purity is critical for the experiments, we recommend immunopanning protocols previously described (Foo et al., 2011).

## Support Protocol 1 PREPARATION OF POLY‐d‐LYSINE–COATED COVERSLIPS

It is important to etch and clean the coverslips before coating them. Etching, in this case with a strong base, allows the poly‐d‐lysine to adhere to the glass and, consequently, the neurons to attach. Cleaning removes dust and debris from the glass, which is optimal for imaging. Coverslips can be prepared in advance; however, we found that coating the coverslips with poly‐d‐lysine overnight immediately prior to dissection (Basic Protocol 1) yields the healthiest neuron cultures.

### Materials

Potassium hydroxide (KOH; Thermo Fisher Scientific, p250‐500)
Distilled pure water
100% ethanol (200 proof; Thermo Fisher Scientific, A4094)
20% (v/v) ethanol in distilled water
Dental wax (paraffin; color‐free, fragrance‐free)
20× poly‐d‐lysine stock solution [1 mg/ml poly‐d‐lysine hydrobromide (Sigma‐Aldrich, P6407) in DPBS; store in 1‐ml aliquots at −20°C]
DPBS (Ca/Mg‐free; Life Technologies, 14190‐235)

1‐L glass bottle
25‐mm round glass coverslips (Electron Microscopy Sciences, 72196‐25)
Ceramic coverslip holders (Thermo Fisher Scientific, 8542E40)
Forceps (nonsterile and sterile)
Nalgene jars (straight‐sided, wide‐mouth polycarbonate jars; Thermo Fisher Scientific, 2116‐0125)
Ultrasonic water bath
Razor blade
60‐mm or 10‐cm culture plate
6‐well or 35‐mm culture dish
Sterile syringe with Luer‐Lok tip (BD Biosciences, 302832)
0.20‐µm filter (Corning, 431219)
37°C cell culture incubator

### Etch and clean coverslips

1 Prepare 1 M KOH solution by dissolving 56 g KOH in 1 L distilled pure water in a 1‐L glass bottle.
  Unused KOH solution can be stored at room temperature and used later.
2 Place individual 25‐mm round glass coverslips in ceramic coverslip holders using forceps and place coverslip holders in Nalgene jars.
3 Fill jars with enough 1 M KOH to fully submerge the coverslips (∼100 ml). Take care to pour KOH solution slowly so as to not dislodge the coverslips from the holders.
4 Place jars in an ultrasonic water bath, with the lids slightly open to allow airflow. Fill bath with enough water to be level with the KOH in the jars. Sonicate for 1 hr on high.
  Do not fill the bath past the jar lid, as this will risk contaminating the coverslips with nonsterile bath water.
5 Rinse coverslips three times with distilled pure water by removing the ceramic holders containing coverslips from the jars, emptying the jars, refilling the jars with fresh distilled pure water, and submerging the holders containing coverslips in the fresh water.
6 Fill jars with 100% ethanol to fully submerge the coverslips (∼100 ml).
7 Place jars in the ultrasonic water bath, with lids slightly open to allow airflow, and sonicate for 1 hr on high.
8 Remove ceramic holders containing coverslips, pour out ethanol, and refill the jars with 20% ethanol in distilled pure water to fully submerge the coverslips. Close lids tightly.
9 Store coverslips in 20% ethanol solution at 4°C until ready for use. When ready, open jars in a sterile culture hood and remove coverslips with sterile forceps to prevent contamination of the coverslips.

### Adhere wax spacers on coverslips for neuronal cultures

**Figure 2:**
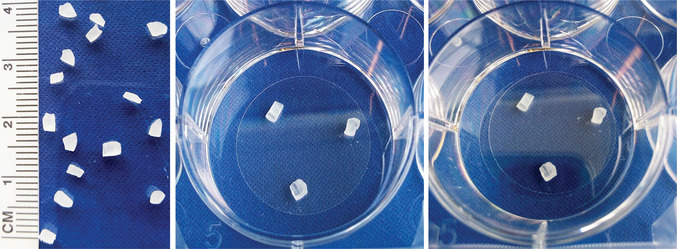
Fatty acid transfer assay setup. Left panel: Wax spacer size. Middle panel: Coverslip with wax spacers attached. Right panel: Second coverslip placed on top of the first coverslip to make a “sandwich.”

10 Using a razor blade, cut dental wax into square spacers (1 × 2 × 3 mm or smaller; Fig. 2, left panel).

11 Place wax spacers in a 60‐mm or 10‐cm cell culture plate and sterilize wax by submerging in 100% ethanol for 5 min. Aspirate ethanol in a sterile culture hood and let wax pieces air‐dry completely.
12 The day before culturing cells, using sterile forceps, place cleaned coverslips (see step 9) in the wells of a 6‐well or 35‐mm culture dish and let dry thoroughly. Aspirate any remaining ethanol to speed the drying process.
13 Using sterile forceps, place three wax spacers (see step 11) on dry coverslips in a triangular pattern. Press gently so that wax adheres to the glass (Fig. 2, middle panel).
  Prepare coverslips with wax spacers for neurons. Astrocytes will be cultured on coverslips without spacers. Placing spacers on neuronal coverslips prevents mechanical damage to the neurons when the coverslips are placed facing each other. Be very careful from this point on not to dislodge the wax spacers from the glass.

### Coat coverslips with poly‐d‐lysine

14 Prepare 1× poly‐d‐lysine working solution by diluting 1 ml of 20× poly‐d‐lysine stock solution in 19 ml DPBS. Filter solution using a sterile syringe with a Luer‐Lok tip and 0.20‐µm filter.
  Sterile working solution can be stored for ≤1 month at 4°C.
15 Add 250 µl of 1× poly‐d‐lysine working solution to center of each coverslip. Allow poly‐d‐lysine to spread over the surface of the coverslip. Put lid back on the cell culture dish and incubate coverslips a 37°C cell culture incubator for ≥2 hr (preferably overnight).
  Surface tension will keep the poly‐d‐lysine on the coverslips, without spreading to the plastic dish. Be careful not to bump the dish to avoid causing the poly‐d‐lysine to spill onto the plastic. If this happens, fewer cells will grow on the coverslips.
16 Wash coverslips three times with 2 ml DPBS and proceed to Basic Protocol 1 (add desired medium in step 3 and proceed with plating cells in step 27).

## Basic Protocol 2 LIPID TRANSFER ASSAY

**Figure 3:**
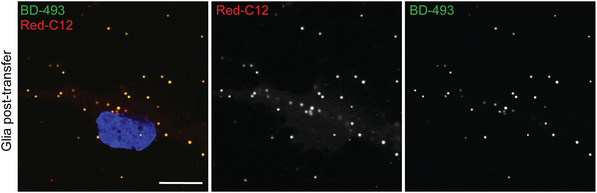
Appearance of neuron‐derived fatty acids (Red‐C12) in glial lipid droplets. After the transfer assay, glia were fixed, stained with BODIPY 493/503 (BD‐493) to label lipid droplets, and imaged using a Zeiss 880 confocal microscope with a 63× objective. Scale bars are 10 µm.

This protocol describes how to use “sandwich”‐style co‐cultures to assay lipid transfer from neurons to glia. This protocol specifically follows the trafficking itinerary of the fluorescently labeled, saturated fatty acid analog BODIPY 558/568 C12 (Red‐C12) into lipid droplets labeled with BODIPY 493/503 (Fig. 3). However, it is possible that this protocol could be used to investigate the trafficking of other fluorescently labeled lipids.

### Materials

HBSS (phenol red–free, Ca/Mg‐free; Thermo Fisher Scientific, 14175095)
1 M HEPES (Thermo Fisher Scientific, 15630106)
Calcium chloride
BODIPY 558/568 C12 (Red‐C12; Thermo Fisher Scientific, D3835)
Dimethylsulfoxide (DMSO)
16% (v/v) paraformaldehyde (Electron Microscopy Sciences, 15710‐S)
DPBS (Ca/Mg‐free; Life Technologies, 14190‐235)
Neuron medium (see recipe)
Plating/glia medium (see recipe)
Neurons on glass coverslips (see Basic Protocol 1)
Glia on glass coverslips (see Basic Protocol 1)
BODIPY 493/503 (Thermo Fisher Scientific, D3922)
Mounting medium (Vectashield Hardset Antifade Mounting Medium with DAPI, Vector Laboratories, H‐1500)
Lens cleaner or 70% (v/v) ethanol

Sterile forceps
Tinfoil
Microscope slide (Thermo Fisher Scientific, 112‐550‐123)
Kimwipes
Confocal or widefield microscope with 63× objective

*NOTE*: All solutions and equipment coming into contact with cells must be sterile, and proper sterile technique should be used accordingly.

*NOTE*: All culture incubations are performed in a humidified 37°C, 5% CO_2_ incubator unless otherwise specified.

### Prepare reagents for transfer assay

1 HBSS plus calcium: Combine 500 ml HBSS, 5 ml of 1 M HEPES (10 mM final concentration), and 0.111 g calcium chloride (2 mM final concentration). Warm to 37°C before use.
  Addition of calcium to a final concentration of 2 mM is required for neuronal activity.
  If storage is necessary, store at 4°C.
2 5 mM Red‐C12 stock solution: Dissolve 1 mg Red‐C12 in 425 µl DMSO.
  If storage is necessary, store at −20°C and warm to room temperature before use.
3 3% (v/v) paraformaldehyde: Combine 187 µl of 16% (v/v) paraformaldehyde with 813 µl DPBS.
  Store and use at room temperature.
4 Warm DPBS, neuron medium, and plating/glia medium to 37°C before use.

### Transfer assay

5 One day prior to the assay, load neurons on glass coverslips with Red‐C12 by replacing half of the medium with 1 ml fresh pre‐warmed neuron medium (leaving 1 ml neuron‐conditioned medium) containing 1 µl of 5 mM Red‐C12 (see step 2; 2.5 µM final concentration).
6 Approximately 18 hr later, wash neurons two times with 2 ml pre‐warmed DPBS and incubate neurons in 2 ml fresh neuron medium for 1 hr at 37°C.
7 Wash neurons and glia on glass coverslips two times with 2 ml DPBS.
8 Add 1 ml pre‐warmed HBSS plus calcium (see step 1) to glia. Use sterile forceps to lift coverslips carrying neurons and place them face‐down onto coverslips carrying astrocytes (Fig. 2, right panel).
  The dental wax spacers will keep the coverslips from directly touching.
9 Incubate sandwich cultures for 4 hr at 37°C.
10 Carefully remove each neuronal coverslip using forceps.
  If the neurons are to be imaged, remove the wax spacers from the coverslips prior to mounting.
11 To fix the glial cells, wash coverslips two times with 2 ml DPBS, fix cells for 10 min in 1 ml of 3% paraformaldehyde (see step 3; protect from light using tinfoil), and wash two times with DPBS.

### Stain for lipid droplets with BODIPY 493/503

12 To make a 5 mg/ml BODIPY 493/503 stock solution, dissolve 10 mg BODIPY 493/503 in 2 ml DMSO.
  If storage is necessary, store at −20°C and warm to room temperature before use.
13 To make a 5 µg/ml BODIPY 493/503 working solution, dilute 5 mg/ml stock solution 1:1000 in DPBS.
14 Incubate fixed glial cells from step 11 in 1 ml of 5 µg/ml BODIPY 493/503 working solution for 10 min at room temperature (protect from light using tinfoil) and wash three times with 2 ml DPBS.
  Staining the recipient glial cells with BODIPY 493/503 serves to verify the presence of lipid droplets; this is where the majority of internalized fatty acids will be stored. In the absence of this stain, if no fatty acids were observed in the recipient cells, it would be impossible to conclude whether there was no transfer or whether the internalized fatty acids were too dispersed (for instance, on membranes) to detect.
  This step can be modified to answer more specific questions: for example, whether transferred fatty acids associate with other organelles, such as mitochondria or peroxisomes.

### Mount and image coverslips

15 Place a drop of mounting medium on a microscope slide.
16 Using forceps, submerge a coverslip carrying fixed glial cells (see step 14) in water and touch side of the coverslip to a Kimwipe to remove excess water.
17 Place coverslip gently onto the drop of mounting medium (see step 15), with the cells facing down. Be careful to avoid trapping bubbles.
18 Gently aspirate excess mounting medium from sides of the coverslip. Be careful not to move or twist coverslip, as this can damage the cells. If present, leave excess mounting medium on top of the coverslip in place for now. Cover to protect from light and let dry overnight at room temperature. Store mounted cells at 4°C protected from light until ready to image.
19 Before imaging, gently clean surface of the coverslip using a Kimwipe and lens cleaner or 70% ethanol.
20 Image coverslip using a confocal or widefield microscope with a 63× objective. To choose regions to image in a non‐biased manner, select healthy cells using DAPI channel.
  Be consistent with the density of nuclei per field of view. Clustering of cells can influence the number of lipid droplets. We typically chose fields of view with 2 to 3 nuclei.
  If a widefield microscope is used, it is critical to perform background subtraction (see Support Protocol 4).
21 Acquire a Z‐stack with 0.5‐µm step size per field of view.
  We typically acquire 10 images per coverslip. At least one image of the DAPI staining is necessary for quantification.

## Support Protocol 2 IMMUNOSTAINING WITH CELL TYPE–SPECIFIC ANTIBODIES

**Figure 4:**
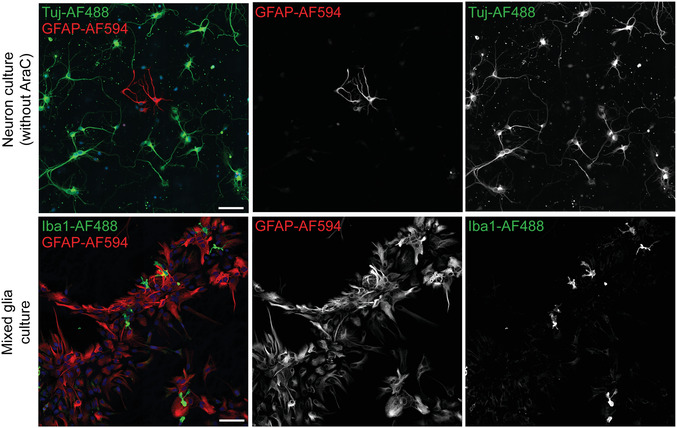
Immunostaining of different cell types in culture. Neuronal cultures in the absence of AraC and mixed glial cultures were immunostained for the neuronal marker tubulin β3 (Tuj), astrocyte marker GFAP, or microglia marker Iba1. Cells were imaged using a Zeiss 880 confocal microscope with a 20× objective. Tiling of 2 × 2 was used, with 10% overlap. Scale bars are 100 µm.

**Figure 5:**
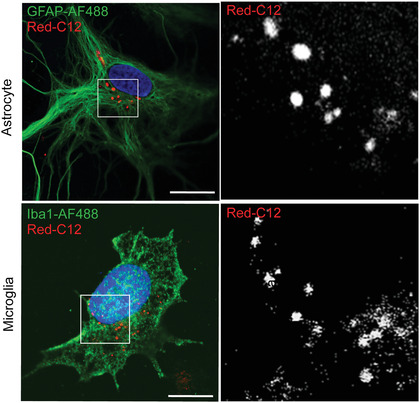
Immunostaining of astrocytes and microglia after transfer assay. After the transfer assay, glia were fixed and immunostained with anti‐GFAP and anti‐Iba1 to label astrocytes and microglia, respectively. Cells were imaged using a Zeiss 880 confocal microscope with a 63× objective. Scale bars are 10 µm.

As primary cultures often contain multiple cell types (mixed glial cultures), it may be useful to stain with antibodies to identify the cell types involved in the transfer assay (Basic Protocol 2). Here, we describe a protocol for immunostaining with antibodies that recognize neurons, astrocytes, and microglia (Fig. 4). This allows assessment of transfer of Red‐C12 into different cell types (Fig. 5). In this particular protocol, the secondary antibodies were selected in order to perform four‐color imaging, including imaging of the Red‐C12 from Basic Protocol 2. This protocol can be adapted to include BODIPY 493/503, as described above, or to stain for oligodendrocytes and organelle‐specific markers with appropriate antibodies.

### Materials

Cells on glass coverslips (mixed glial cells or neurons; see Basic Protocol 2)
Blocking buffer: 2% (w/v) bovine serum albumin (BSA; Sigma‐Aldrich, B4287) and 0.2% (v/v) Triton X‐100 (Thermo Fisher Scientific, BP151‐100) in PBS
Chicken anti‐GFAP (Abcam, ab4674; RRID:AB_304558)
Rabbit anti‐Iba1 (Wako, 019‐19741; RRID:AB_839504)
Mouse anti–tubulin β3 (TuJ1; BioLegend, 801202; RRID:AB_10063408)
Wash buffer: 0.1% (v/v) Triton X‐100 (Thermo Fisher Scientific, BP151‐100) in PBS (stored at room temperature in sealed container)
Alexa Fluor 647–labeled goat anti‐chicken (Thermo Fisher Scientific, A‐21449; RRID:AB_2535866)
Alexa Fluor 488–labeled donkey anti‐rabbit (Thermo Fisher Scientific, A‐21206; RRID:AB_2535792)
Alexa Fluor 488–labeled goat anti‐mouse (Thermo Fisher Scientific, A‐11001; RRID:AB_2534069)
Mounting medium (Vectashield Hardset Antifade Mounting Medium with DAPI, Vector Laboratories, H‐1500)

Orbital platform shaker
Microscope slide (Thermo Fisher Scientific, 112‐550‐123)
Forceps
Kimwipes

Additional reagents and equipment for cell fixation and imaging (see Basic Protocol 2)

### Fix and stain cells

1 Fix cells with paraformaldehyde, as described in Basic Protocol 2, step 11.
2 Block and permeabilize cells by incubating in 1 ml blocking buffer for 1 hr at room temperature while shaking gently on an orbital platform shaker.
3 Prepare primary antibodies: For mixed glial cultures, dilute 1 µl anti‐GFAP and 1 µl anti‐Iba1 in 750 µl blocking buffer per coverslip. For neuron cultures, dilute 1 µl TuJ1 and 1 µl anti‐GFAP in 750 µl blocking buffer per coverslip.
4 Incubate cells with primary antibodies for 1 hr at room temperature or overnight at 4°C while shaking gently on an orbital shaker.
  If incubating overnight, the orbital shaker can be placed in a cold room.
5 Wash three times with 2 ml wash buffer, 15 min each time, at room temperature while shaking gently on an orbital shaker.
6 Prepare secondary antibodies: For mixed glial cultures, dilute 1 µl Alexa Fluor 647–labeled goat anti‐chicken and 1 µl Alexa Fluor 488–labeled donkey anti‐rabbit in 750 µl blocking buffer per coverslip. For neuron cultures, dilute 1 µl Alexa Fluor 647–labeled goat anti‐chicken and 1 µl Alexa Fluor 488–labeled goat anti‐mouse in 750 µl blocking buffer per coverslip.
  If multiplexing with BODIPY 493/503 is desired, no cell type–specific marker can be used in the green channel (in this case, Alexa Fluor 488). When combining immunostaining with BODIPY 493/503 staining, we add 5 µg/ml BODIPY 493/503 along with the secondary antibodies in blocking buffer.
  We do not recommend directly comparing quantifications of BODIPY 493/503 when incubated for 1 hr with quantifications based on the 10‐min protocol described in Basic Protocol 2. This is because longer staining times may stain lipid droplets more strongly, making them appear larger or possibly revealing small lipid droplets that were not detected after 10 min.
7 Incubate cells with secondary antibodies for 1 hr at room temperature while shaking gently on an orbital shaker.
8 Wash three times with 2 ml wash buffer, 15 min each time, at room temperature while shaking gently on an orbital shaker.

### Mount and image coverslips

9 Place a drop of mounting medium on a microscope slide.
10 Using forceps, submerge a coverslip carrying fixed cells (see step 8) in water and touch side of the coverslip to a Kimwipe to remove excess water.
11 Place coverslip gently onto the drop of mounting medium (see step 9), with the cells facing down. Be careful to avoid trapping bubbles.
12 Gently aspirate excess mounting medium from sides of the coverslip. Be careful not to move or twist the coverslip as this can damage the cells. If present, leave excess mounting medium on top of the coverslip in place for now. Cover to protect from light and let dry overnight at room temperature. Store mounted cells at 4°C protected from light until ready to image.
13 Before imaging, gently clean surface of the coverslip using a Kimwipe and lens cleaner or 70% ethanol.
14 Image cells as described above (see Basic Protocol 2, steps 20 and 21).

## Support Protocol 3 CONTROLLING FOR PHAGOCYTOSIS AND TRANSFER OF CELL DEBRIS

Phagocytosis is an important mechanism that astrocytes and microglia use to take up debris and dying cells following injury. Phagocytosis of cell debris containing membranes labeled with fluorescently tagged fatty acids can contribute to fatty acid transfer and lipid droplet formation. Therefore, it is important to distinguish between fatty acid transfer due to phagocytosis and transfer mediated by lipid particles. Here, we outline two control experiments to quantify the contribution of phagocytosis of cell debris in the fatty acid transfer assay (Basic Protocol 2): a cell filtration protocol and a centrifugation protocol. These strategies can be used in parallel to the experiments described in Basic Protocol 2. Generally, neurons are loaded with Red‐C12, and then after washing, they are placed in HBSS plus calcium instead of in a sandwich culture. After an incubation period equivalent to that of the transfer assay (Basic Protocol 2), the HBSS plus calcium is removed from the neurons. Glial cells are then incubated either with the conditioned HBSS plus calcium or with conditioned HBSS plus calcium that has been filtered or centrifuged; as cellular debris and apoptotic bodies shed from dying neurons are roughly 0.8 to 5 µm in diameter, they can be removed by either filtration or centrifugation (Crescitelli et al., 2013).

### Additional Materials (also see Basic Protocol 2)

2‐ml microcentrifuge tubes
0.20‐µm filter (Corning, 431219)
3.5‐ml polycarbonate ultracentrifugation tubes (Beckman Coulter, 362305)
Beckman Coulter Optima MAX‐TL centrifuge and TLA‐110 fixed‐angle rotor
37°C water bath

*NOTE*: All solutions and equipment coming into contact with cells must be sterile, and proper sterile technique should be used accordingly.

*NOTE*: All culture incubations are performed in a humidified 37°C, 5% CO_2_ incubator unless otherwise specified.

### Prepare cells

1 Approximately 18 hr prior to the assay, load neurons on glass coverslips with Red‐C12 by replacing half of the medium with 1 ml fresh neuron medium (leaving 1 ml neuron‐conditioned medium) containing 1 µl of 5 mM Red‐C12 (see Basic Protocol 2, step 2; 2.5 µM final concentration). For the filtration protocol, prepare at least two coverslips or for the centrifugation protocol, prepare three coverslips.
2 Wash neurons two times with 2 ml pre‐warmed DPBS and incubate neurons with 2 ml fresh neuron medium for 1 hr at 37°C.
3 Wash neurons two times with 2 ml DPBS.
4 Add 1.5 ml HBSS plus calcium (see Basic Protocol 2, step 1) to each neuronal coverslip and incubate for 4 hr at 37°C.
5 Proceed to either filtration protocol (steps 6a to 10a) or centrifugation protocol (steps 6b to 13b).

### Filtration protocol

6a Collect neuron‐conditioned HBSS from two coverslips into two separate 2‐ml microcentrifuge tubes.
7a Filter one sample with a 0.2‐µm filter.
8a Wash two coverslips carrying glia two times with 2 ml pre‐warmed DPBS.
9a To one glial coverslip, add 1 ml unfiltered neuron‐conditioned HBSS (control; see step 6a), and to the second glial coverslip, add 1 ml filtered neuron‐conditioned HBSS (see step 7a). Then, incubate cells for 4 hr at 37°C.
10a Fix, mount, and image cells as described in Basic Protocol 2, step 11 and steps 15 to 21.

### Centrifugation protocol

6b Collect neuron‐conditioned HBSS from three coverslips into separate 3.5‐ml polycarbonate ultracentrifugation tubes.
7b Set one tube on ice, protected from light (control).
8b Centrifuge second tube for 20 min at 15,000 × *g*. Transfer supernatant to a clean ultracentrifugation tube and store on ice protected from light.
  This low‐g sample is depleted of apoptotic bodies and cell debris.
9b Centrifuge third tube for 2.5 hr at 250,000 × *g*. Transfer supernatant to a clean ultracentrifugation tube and store on ice protected from light.
  This high‐g sample is depleted of lipid particles and extracellular vesicles in addition to apoptotic bodies and cell debris.
10b Briefly warm control conditioned HBSS (see step 7b) and low‐*g* (see step 8b) and high‐*g* (see step 9b) supernatants in a 37°C water bath.
11b Wash three coverslips carrying glia two times with 2 ml pre‐warmed DPBS.
12b Add control‐conditioned HBSS or low‐*g* or high‐*g* supernatant to glial coverslips and incubate for 4 hr at 37°C.
13b Fix, mount, and image cells as described in Basic Protocol 2, step 11 and steps 15 to 21.

## Support Protocol 4 ANALYSIS OF LIPID DROPLETS WITH ImageJ

**Figure 6:**
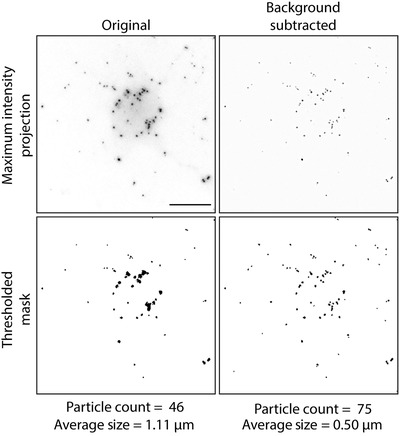
Red‐C12 detection to quantify fatty acid transfer. Thresholding in the absence of background subtraction can give inaccurate particle counts. Lipid droplets clustered together in the perinuclear region may be counted as one large lipid droplet, and smaller peripheral lipid droplets are not detected at all. Subtracting a Gaussian blurred duplicate of the original image removes the background, allowing for more accurate particle detection. Depicted here are maximum intensity projections of Z‐stacks with 0.5‐µm step size acquired using a Nikon Eclipse TiE widefield microscope with a 60× objective. Scale bars are 10 µm.

Here, we describe a protocol for analyzing transfer of labeled fatty acids into lipid droplets (Basic Protocol 2) using ImageJ, an open‐source image‐processing program. These experiments can be performed with a range of imaging modalities. We typically use confocal imaging to analyze co‐localization due to superior contrast and widefield imaging with an electron‐multiplying CCD (EMCCD) camera to collect images for quantification due to greater sensitivity and speed. Background subtraction is especially important if images are collected using a widefield microscope; this allows more accurate thresholding for particle detection (Fig. 6).

Useful tip: You can automate your analysis in ImageJ without knowledge of programming. In the Plugins menu, click on Macros and then select Record before analyzing one image. After you have completed the analysis, hit the Create button to save the code written by ImageJ.

### Materials

ImageJ (RRID:SCR_003070) or Fiji (RRID:SCR_002285)
Image Z‐stacks (see Basic Protocol 2 and Support Protocols 2 and 3)

### Remove background from image

1 In ImageJ or Fiji, select an image Z‐stack.
2 Select Image > Stacks > Z‐project > maximum intensity projection (MIP).
3 To create MIP2, select Image > Duplicate.
4 Select MIP2, Process > Filters > Gaussian Blur.
  Sigma (Radius): 2.0 works for most images.
5 To create a background‐subtracted image, select Process > Image Calculator. Input Image 1: MIP1, operation: subtract and Image 2: MIP2 (blurred image).

### Analyze lipid droplet number and size

6 Select background‐subtracted image.
7 Select Image > Adjust > Threshold.
8 Select dark background.
9 Adjust threshold so that lipid droplets are highlighted in red, either manually by using the sliding bars or using the custom thresholding algorithms.
  It is not necessary to press Apply.
  The Otsu, Yen, and Intermodal algorithms work well for detecting lipid droplets. The most important consideration, however, is that the same thresholding method is used consistently when comparing images from different treatment groups.
10 Select Analyze > Particles. Set size to “2‐infinity pixel units” to remove single pixels. Check Summarize box. Click OK.
  This reduces the likelihood of noise being detected as a particle.
  It can be helpful to show Masks to visualize what is being included/excluded in your analysis so that you may modify your parameters accordingly.
11 Save data directly from the Summary window or copy and paste data into an Excel file.
  Image analysis results will be added sequentially to the Summary window without erasing the previous analysis.

### Quantify number and size of nuclei

12 To set measurements, select Analyze > Set Measurements.
13 Select Area and then select OK.
14 Select DAPI image.
15 Select Image > Adjust > Threshold.
16 Select dark background.
17 Adjust threshold so that nuclei are highlighted in red, either manually by using the sliding bars or using the custom thresholding algorithms.
  It is acceptable to have some background pixels detected after thresholding.
18 Select Analyze > Particles. Set size to “25‐infinity pixel units” to remove single pixels. Check Summarize box. Click OK.
  This reduces the likelihood of noise being detected.
  It can be helpful to show Masks to visualize what is being included/excluded in your analysis so that you may modify your parameters accordingly.
19 Save data directly from the Summary window or copy and paste data into an Excel file.
  Image analysis results will be added sequentially to the Summary window without erasing the previous analysis.
  Calculating the number of nuclei can be done manually if the number of cells per image (using a 63× objective) is small (2 to 4 cells). If the experiment is modified such that the number of nuclei is larger, then automated nuclei counting using ImageJ can be more time efficient and accurate. This method is, however, required for evaluating the size of nuclei to determine whether a specific treatment affected cell health.

### Calculate number of lipid droplets per cell

**Figure 7:**
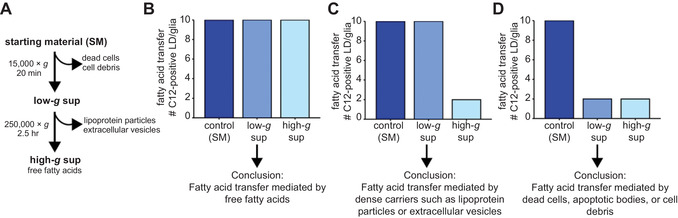
Interpreting centrifugation‐based control assay. (**A**) Centrifugation can be used to selectively remove components from the medium of neurons loaded with fluorescently labeled fatty acids. Recipient cells (glia) are treated with depleted supernatant (sup) and analyzed for the appearance of labeled fatty acids. This schematic is reproduced from Ioannou et al. (2019). (**B**) If centrifugation does not affect the appearance of labeled fatty acids in glia, then fatty acid transfer is mediated by free fatty acids. (**C**) If fatty acid transfer is reduced by high‐*g* centrifugation only, then dense carriers such as lipoprotein particles or extracellular vesicles mediate the transfer. (**D**) If fatty acid transfer is reduced by both high‐*g* and low‐*g* centrifugation, then dead cells, cell debris, and apoptotic bodies mediate the transfer.

20 To calculate the number of Red‐C12‐positive particles per cell, divide number of Red‐C12‐positive particles detected (see step 11) by number of nuclei (see step 19).
21 If the filtration protocol was performed (see Support Protocol 3), compare number of Red‐C12‐positive particles in the control sample versus that in the filtered sample.
  If there is no difference between the samples, phagocytosis of cell debris can be excluded.
22 If the centrifugation protocol was performed (see Support Protocol 3), compare number of Red‐C12‐positive particles in the control sample versus low‐*g* and high‐*g* supernatants.
  If there is a reduction in the number of particles following low‐g centrifugation, fatty acids are being transferred by cell debris. If there is a reduction in the number of particles following high‐g centrifugation, small, dense carriers such as extracellular vesicles or lipoprotein particles are mediating fatty acid transfer. If there is no difference in the number of particles detected after either low‐g or high‐g centrifugation, free fatty acids are mediating transfer. See Fig. 7.

23 As a secondary measure of cell health, compare mean nuclei size and number between treatment groups.
  If differences between groups are observed, then alterations in cell health may be influencing the results and should be considered when interpreting all results.

## REAGENTS AND SOLUTIONS

### Blocking buffer

Add 1 g bovine serum albumin (BSA; Sigma‐Aldrich, B4287) to 50 ml PBS and shake (vortex or rocking) until completely dissolved. Filter solution using a sterile syringe with a Luer‐Lok tip (BD Biosciences, 302832) and 0.20‐µm filter (Corning, 431219). Add 1 ml 10% (v/v) Triton X‐100 (Thermo Fisher Scientific, BP151‐100). This will give final concentrations of 2% (w/v) BSA and 0.2% (v/v) Triton X‐100. Store overnight at 4°C. Make fresh for each experiment.

### Dissection medium

Combine 500 ml HBSS (Ca/Mg free; Thermo Fisher Scientific, 14175095), 5 ml 1 M HEPES (pH 7.4; Thermo Fisher Scientific, 15630106; 10 mM final), 5 ml 100 mM sodium pyruvate (Thermo Fisher Scientific, 11360070; 1 mM final), and 2.5 ml 20% (w/v) glucose (see recipe; 0.1%; final). Store ≤6 months at 4°C.

### Glucose, 20%

Add 10 g d‐glucose (Thermo Fisher Scientific, D16‐500) to 50 ml ddH_2_O and mix until fully dissolved. It can be helpful to warm the solution and/or use a vortex. Filter solution using a sterile syringe with a Luer‐Lok tip (BD Biosciences, 302832) and 0.20‐µm filter (Corning, 431219). Store ≤6 months at 4°C.

### Neuron medium

Use NbActiv4 medium (BrainBits, Nb4‐500), which already contains the supplements needed to support neuronal growth. Optionally, add 5 ml 100× antibiotic‐antimycotic (Thermo Fisher Scientific, 15240096; 1× final) to 500 ml NbActiv4 to protect cells from contamination. Store ≤2 months at 4°C or according to expiry date listed by the manufacturer.

### Plating/glia medium

Combine 500 ml Basal Medium Eagle (BME) with Earle's salts (Invitrogen, 21010046), 50 ml fetal bovine serum (FBS; heat inactivated; Gibco, 16000036; 10% final), 5 ml 100 mM sodium pyruvate (Thermo Fisher Scientific, 11360070; 1 mM final), 5 ml 100× GlutaMAX (Thermo Fisher Scientific, 35050‐061; 1× final), and 12.5 ml 20% (w/v) glucose (see recipe; 0.45% final). Optionally, add 5 ml 100× antibiotic‐antimycotic (Thermo Fisher Scientific, 15240096; 1× final) to protect cells from contamination. Store ≤2 months at 4°C.

## COMMENTARY

### Background Information

The ability of glia to supply neurons with lipids and cholesterol has been appreciated for many decades. Astrocytes and microglia synthesize and secrete lipoprotein particles composed of a cholesterol and neutral lipid core surrounded by a phospholipid monolayer and apolipoproteins. Neurons use the lipids and cholesterol from glia‐derived lipoprotein particles in order to build membranes and form synapses (Holtzman et al., 1995; Mauch et al., 2001). There are many challenges associated with studying lipid transport in the brain or in neuron‐glia co‐cultures. Lipids cannot be targeted by the same genetic tools commonly used for studying proteins, such as tagging with fluorescent proteins in a cell type–specific manner. Although numerous fluorescently tagged lipids are commercially available, there are limitations to use of exogenously applied lipids when studying cells of the nervous system. For example, fatty acid transport has been demonstrated between fibroblasts by co‐culturing donor cells (loaded with fluorescent fatty acids) with acceptor cells in the same dish (Rambold, Cohen, & Lippincott‐Schwartz, 2015); neurons and glia, however, need to be grown in different types of medium to facilitate their differentiation and survival and require several days to mature. Phagocytosis also becomes a confounding issue, as primary cells have more cell death upon initial plating compared to immortalized cell lines. These factors preclude the ability to perform fatty acid transfer assays in mixed neuron‐glial cultures as previously described. Factors secreted by astrocytes and detected by neurons can be probed using a “sandwich” co‐culture system (Jones, Cook, & Murai, 2012). Combining the “sandwich” co‐culture system with the lipid transfer assay overcomes the difficulties associated with primary neuron cultures and allows investigation of lipid trafficking between neurons and glia. Using this lipid transfer assay, we recently discovered that neurons also transfer lipids to astrocytes and microglia (Ioannou et al., 2019). This transfer is dependent on neuronal expression of ApoE, but much remains to be discovered regarding the mechanisms of lipid transport from neurons to astrocytes. The described lipid transfer assay will allow these mechanisms to be dissected in order to gain a fuller understanding of how neurons and astrocytes couple their lipid metabolism.

### Critical Parameters

As with all experiments involving primary neuron cultures, the health of the neurons is critical. If neurons are unhealthy, they may release lipids via apoptotic bodies or via debris from dead cells, which will interfere with specific experiments. Testing for the mode of transport will determine whether this is a factor (Support Protocol 3). Another important consideration is the purity of the neuronal culture. As glia secrete lipoprotein particles that contribute to lipid transfer, glial contamination needs to be minimal to avoid misinterpretation of results. We recommend always keeping the percentage of contaminating glia to <2% (Support Protocol 2).

### Troubleshooting

Please refer to Table 1 for a troubleshooting guide.

**Table 1:** Troubleshooting Guide for Neuron‐Glia Co‐culture for Studying Intercellular Lipid Transport

Problem	Possible cause	Solution
Too much cell death when plating	Trituration is too harsh. Papain concentrations are too low. Duration between removing tissue and plating cells is too long.	Triturate gently and make sure no air bubbles are present. Increase amount of papain used. Reduce number of animals dissected until technical skills are refined. Ideally, cells should be in incubator within 1‐1.5 hr of euthanizing animal.
Cell culture contamination	More sterile culture technique is needed.	Sterilize dissection tools in autoclave. Clean all working surfaces with 70% ethanol.
Cells not adhering to coverslips	Poly‐d‐lysine coating is inadequate.	Make fresh poly‐d‐lysine. Coat etched coverslips overnight and use within 24 hr.
Cell death several days after plating	Neuron medium is old. Full medium changes are used.	Store medium in foil to protect from light. Avoid repeated warming and cooling. Avoid full medium changes, which deprive neurons of secreted neurotrophic factors required for survival. Change only half of the medium every 3‐4 days.
Too many glia in neuron cultures	Pups are too old at time of dissection. Serum is present in cultures. AraC concentration is too low or AraC is added too late.	Use P0 animals. Serum induces differentiation of precursor cells into glia. Change medium completely morning after plating to remove any remaining serum. Add AraC 2 days after plating. It may help to keep AraC in medium indefinitely to prevent glial cell growth.
Too much cell death during transfer assay	Desired treatment causes too much cell death.	Optimize dose and time course.
No transfer	Labeled fatty acids do not accumulate in lipid droplets.	Confirm that glial lipid droplets are present by staining with BODIPY 493/503. Small amounts of BODIPY 558/568 C12 (Red‐C12) can be dim and are sometimes challenging to see through eyepiece. Ensure proper imaging setup (excitation/emission) and adjust gain and laser power. Use microscope with highly sensitive detectors (we typically use Zeiss 880 GaAsp detectors).

### Understanding Results

As described above, phagocytosis of apoptotic bodies and cell debris can contribute to fatty acid transfer and lipid droplet formation (Support Protocol 3). This means that if a desired treatment induces neuronal cell death, more fatty acid transfer will likely be observed. In order to study the mechanisms of lipid particle–mediated transfer of fatty acids, one must carefully control for and monitor cell death. In addition, we include control experiments (Support Protocol 3) to directly test the mechanism of transfer involved. By using low‐*g* and high‐*g* centrifugation, various components can be depleted from neuron‐conditioned medium. If fatty acid transfer is abolished by low‐*g* centrifugation, then apoptotic bodies and cell debris are mediating the transfer. If fatty acid transfer is abolished only by high‐speed centrifugation, then dense carriers such as lipoprotein particles or extracellular vesicles mediate the transfer. Finally, if neither low‐*g* nor high‐*g* centrifugation affects the rates of fatty acid transfer, then the transfer is likely mediated by free fatty acids bound to carrier molecules such as albumin.

Another consideration is that because fluorescently labeled fatty acids accumulate in glial lipid droplets, the presence of glial lipid droplets is essential for detecting and quantifying transfer. Some treatments can influence fatty acid transfer and/or lipid droplet numbers in different ways. For example, neuronal activation using the chemogenetic receptor system hM3D(Gq) increases fatty acid transfer to glia. However, glutamate released by neural activity promotes glial consumption of fatty acids stored in lipid droplets, resulting in a reduction in the number of lipid droplets. Therefore, if glutamate or N‐methyl‐D‐aspartate (NMDA) is added into the transfer assay, one might mistakenly conclude that there is a reduction in fatty acid transfer, when instead fatty acids/lipid droplets are being consumed at a faster rate. Therefore, it is important to analyze both the number of glial lipid droplets containing transferred fatty acids and the total number of lipid droplets.

### Time Considerations

Preparing primary cell cultures (Basic Protocol 1) should take ∼3 hr, including setup. Once the cells are plated, they will require medium changes after 6 hr and 24 hr and then every 3 to 4 days for maintenance. Neurons are ideally used after between 1 and 2 weeks in culture. Astrocytes can be used by 1 week. If older astrocytes are desired, they can be initially plated on culture dishes and split to avoid over‐confluence. For Support Protocol 1, it will take ∼3 hr to clean the coverslips, which can be stored for later use, and coating the coverslips requires an overnight incubation. For lipid transfer assays (Basic Protocol 2), cells are treated with fluorescently labeled fatty acids the night before the assay. On the day of the assay, the labeled neurons are incubated in fresh medium for 1 hr prior to the assay. The sandwich co‐culture takes 4 hr. Following the assay, cells can be fixed for 10 min and mounted. If immunolabeling is performed (Support Protocol 2), this will take an additional 24 hr. Experiments controlling for phagocytosis (Support Protocol 3) take ∼10 hr or ∼14 hr for the filtration and centrifugation assays, respectively. Images can be analyzed (Support Protocol 4) in ∼15 min per coverslip.
